# Does side matter? Deciphering mechanisms that underpin side-dependent pathogenesis and therapy response in colorectal cancer

**DOI:** 10.1186/s12943-025-02327-5

**Published:** 2025-05-02

**Authors:** Harrison J. Boka, Rebekah M. Engel, Christine Georges, Paul J. McMurrick, Helen E. Abud

**Affiliations:** 1 Department of Anatomy and Developmental Biology, Monash University, Clayton, VIC 3800 Australia; 2 Development and Stem Cells Program, Monash Biomedicine Discovery Institute, Monash University, Clayton, VIC 3800 Australia; 3 Department of Surgery, Cabrini Monash University, Cabrini Hospital, Malvern, VIC 3144 Australia

**Keywords:** Colorectal cancer, Sidedness, Right-sided, Left-sided, Molecular biomarkers

## Abstract

Colorectal cancer (CRC) is stratified by heterogeneity between disease sites, with proximal right-sided CRC (RCRC) multifactorial in its distinction from distal left-sided CRC (LCRC). Notably, right-sided tumors are associated with aggressive disease characteristics which culminate in poor clinical outcomes for these patients. While factors such as mutational profile and patterns of metastasis have been suggested to contribute to differences in therapy response, the exact mechanisms through which RCRC resists effective treatment have yet to be elucidated. In response, recent analyzes, including those utilizing whole genome sequencing, transcriptional profiling, and single-cell analyses, have demonstrated that key molecular differences exist between disease sites, with differentially expressed genes spanning a diverse range of cellular functions. Here, we review and contextualize the most recent data on molecular biomarkers found to exhibit discordance between RCRC and LCRC, and highlight candidates for further investigation, including those which present promise for future clinical application. Given the present disparity in survival outcomes for RCRC patients, we expect the prognostic biomarkers presented in our review to be useful in establishing future directions for the side-specific treatment of CRC.

## Clinical trial number

Not applicable

## Background

Colorectal cancer (CRC) is the third most common malignancy worldwide, accounting for 9% of all cancer-related deaths [1, 2]. Tumors arising from the right (RCRC) and left (LCRC) sides of the colon are clinicopathologically distinct and demonstrate differing prognoses, with clinical studies consistently reporting RCRC patients as less responsive to both cytotoxic chemotherapeutics and targeted therapies [3, 4]. In the hope of improving patient outcomes and developing novel therapy options, the biological and molecular characteristics that underpin RCRC and LCRC must be elucidated.

Fundamental differences in the embryological origin of the right and left colon have been implicated in disease pathogenesis and subsequent treatment response. Molecularly, embryonic patterning of the developing colon is determined by the distinct expression of genes including those within the homeobox (HOX) and caudal-related homeobox (CDX) families [5]. Anatomically, the right colon arises from the embryonic midgut and comprises the caecum, ascending colon, hepatic flexure and proximal two thirds of the transverse colon, while the left colon arises from the hindgut and includes the distal third of the transverse colon, the splenic flexure, descending colon, and rectum, although the rectum has been considered to be of further biological distinction [5–7]. Vasculature and lymphatics develop concurrently and similarly service the colon depending on midgut or hindgut origin. Specifically, the midgut-derived right colon is supplied by the superior mesenteric artery and its branches, while the hindgut-derived left colon is supplied by the inferior mesenteric artery and its derivatives [5]. Lymphatic drainage follows the orientation of the arterial anatomy, while venous drainage of the right and left colon occurs through the superior and inferior mesenteric veins respectively [5, 8, 9]. Despite their similar arrangement, the combined vascular anatomy of the right colon displays more variation than that of the left, with notable differences in vessel orientation and the number of middle colic veins frequently differing between individuals [10–12].

In the context of CRC, the anatomical position of right-sided and left-sided tumors with respect to vasculature and lymphatics has been suggested to influence the route through which they metastasize. Specifically, while the liver and peritoneum are the most common sites of metastasis across primary tumor locations [13], the former represents a higher proportion within LCRC, with RCRC more commonly involving the peritoneum [14–16]. As compared to the likely hematogenous spread of liver metastases [13, 17], peritoneal metastases, which represent distinctly poor outcomes for CRC patients [18], are thought to arise, at least in part, by way of the lymphatic system [13, 19], with drainage of the peritoneal cavity expected to include superior mesenteric lymph nodes along the right colon, based on animal studies [20]. Coupled with the increased likelihood of RCRC being of T4 staging [21, 22], whereby disease extends into the visceral peritoneum [23], as well as colectomy enabling the release of potentially metastatic cells into the peritoneal cavity [24], the lymphatic drainage of the right colon only further facilitates the progression of disease within the peritoneum.

Genetically, key drivers of tumor formation have been seen to differ between RCRC and LCRC, though it remains unclear what role anatomical variation plays in initiating or supporting this growth. Pathogenesis of disease originating from the left side of the colon has long been associated with a conventional “adenoma-to-carcinoma sequence”, characterized by microsatellite stability (MSS) and the sequential accumulation of mutations in genes including *APC*,* KRAS* and *TP53* [25, 26]. Meanwhile, RCRC is more commonly associated with the serrated pathway of carcinogenesis, with microsatellite instability (MSI) and mutations in the *BRAF* gene often inciting poorly differentiated lesions of inferior visibility on colonoscope, increasing the difficulty of timely detection [27–31]. In spite of these associations, the carcinogenic pathways driving RCRC and LCRC are not mutually exclusive, with lesions representing both the conventional and serrated pathways observed across disease sites, albeit observed in different frequencies [32], thus aligning with the idea of a mutational continuum [33]. A recent genomic analysis of over 2000 colorectal tumors has provided comprehensive insight into this hypothesis, with disease further subdivided based on the genomic stability of MSS tumors. While the authors indeed associate MSI with RCRC, they reveal MSS disease of the right side as more likely to be genomically stable, with MSS left-sided disease more commonly associated with whole genome duplication and loss of heterozygosity. Meanwhile, their analysis of MSS tumors across disease sites demonstrated a decrease in the mutation of *IDI1*,* ID2*,* KRAS* and *PIK3CA* and an increase in *TP53* mutations in LCRC [34].

**Fig. 1:**
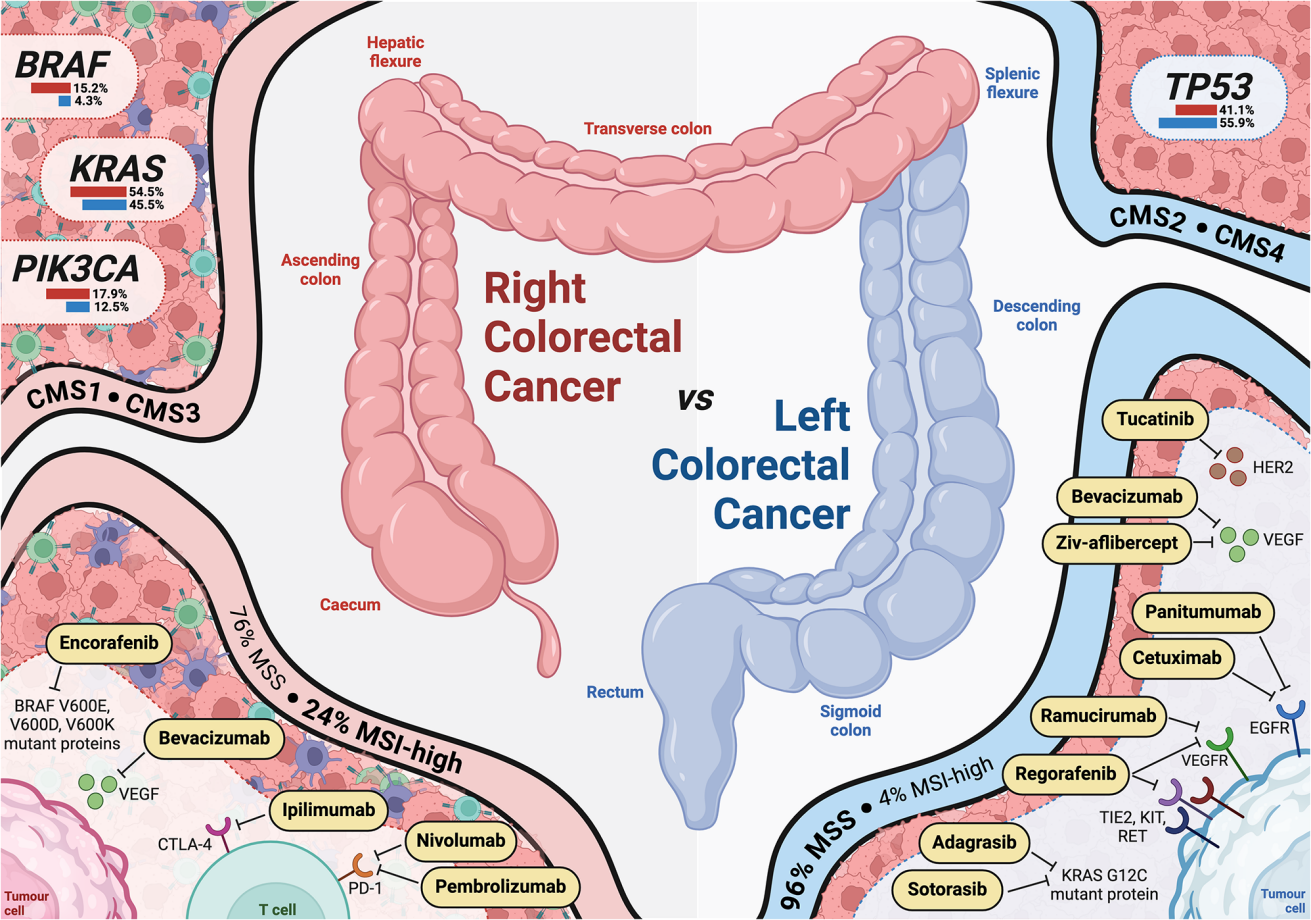
Distribution of clinically relevant biomarkers and approved therapeutic targets between sides. While RCRC is associated with CMS1 and CMS3 profiles, as well as *BRAF*, *KRAS*, and *PIK3CA* mutations, LCRC is associated with CMS2 and CMS4 status, alongside *TP53* mutations. Of the targeted therapies currently approved by the United States Food and Drug Administration for the treatment of CRC, the increased incidence of MSI in right-sided disease dictates RCRC patients as more receptive to immunotherapies, including ipilimumab (anti-CTLA-4), nivolumab (anti-PD-1) and pembrolizumab (anti-PD-1). Such is also the case with mutant forms of the KRAS (G12C) and BRAF (V600E, V600D, V600K) proteins, which favor LCRC and RCRC, respectively. Meanwhile, the increased expression of targets including HER2 (tucatinib), VEGF (bevacizumab, ziv-aflibercept) and EGFR (cetuximab, panitumumab) in LCRC align with the superior response of these patients to the corresponding therapies. Even so, bevacizumab has been demonstrated as more efficacious in RCRC than anti-EGFR therapies or chemotherapy alone, and as such, is recommended for the treatment of both right-sided and *RAS*-mutant disease. Data were retrieved from and based on references [3, 30, 32, 34–36, 38–46]

The distribution of tumor mutational profiles and the identification of actionable driver genes is key to assessing and assigning therapeutic regimes (Fig. 1). Unfortunately, current associations of clinical response with tumor sidedness negate the mutational and transcriptional spectra within which each side of disease lies. For instance, clinical trials assessing the response of patients to anti-EGFR and anti-VEGF therapies display variable results between RCRC and LCRC, with poorer outcomes observed in RCRC patients, both when treated with anti-EGFR therapies and when treated with anti-VEGF therapies. While this has been attributed to the reduced activity of both EGFR and VEGF-1 in right-sided disease [3, 30, 35], the increased prevalence of *KRAS* and *BRAF* mutants in RCRC, suggest these pathways are active in these patients rather than RCRC patients are collectively less responsive to these agents. Regardless, the observation of RCRC as less responsive to anti-EGFR than anti-VEGF therapies [36] is reflected in the European Society for Molecular Oncology (ESMO) Clinical Practice Guidelines for CRC treatment, where RCRC and *RAS*-mutant patients are recommended combination therapies inclusive of bevacizumab (anti-VEGF), while LCRC, *RAS*-wildtype patients are recommended anti-EGFR therapies [37].

While classification systems, such as the consensus molecular subtype (CMS), have been designed to better categorize patients based on tumor and stromal characteristics, they too provide an imperfect method to segregate patient groups and, as intra-tumoral heterogeneity precludes the ascription of a singular phenotype, have yet to make a marked impact on personalized cancer medicine [47, 48]. Furthermore, although RCRC tumors have been seen to align with the CMS1 and CMS3 profiles, and LCRC with CMS2 and CMS4, current targeted therapies are limited to antagonizing a single antigen, rather than the myriad of mutations associated with each CMS subtype, so are incompatible with such a broad approach [33, 46]. Therefore, in order to better tailor side-specific treatment options, we must first deduce the distinct, targetable molecular attributes of right-sided and left-sided disease.

The distinction between RCRC and LCRC is being made progressively clearer by virtue of greater accessibility to whole genome and RNA sequencing, and the subsequent development of large cancer datasets. These datasets, such as the commonly referenced *The Cancer Genome Atlas*, enable researchers to construct high confidence estimations of transcriptional and translational relationships, as well as to simply define genes that are differentially expressed between two conditions [49–54]. Additionally, independent datasets compiled from institutional patient tissue repositories provide further opportunity for discovery, whilst are also commonly combined with publicly available datasets for more robust and comprehensive analyses [55]. Accordingly, this review aims to encapsulate recent advances in the molecular characterization of right and left CRC, focusing primarily on observed differences in gene and protein expression.

### Transcriptional regulators linked to poor clinical outcomes are commonly associated with RCRC

Perturbed gene expression promotes cancer initiation and drives progression [56]. As transcriptional regulators contribute to the maintenance of a neoplastic cell state, key genomic and proteomic instigators have been studied both for their influence on genetic reprogramming, and their ability to be targeted in pursuit of restoring transcriptional homeostasis [57]. Epigenetic modifiers and nucleic acid binding proteins, including transcription factors, are proteins of functional relevance, while certain forms of RNA, including long non-coding RNAs (lncRNAs) and micro RNAs (miRNAs), have also been implicated in cancer-associated gene regulation. This has been further deciphered with the advent of single-cell (scRNAseq) and spatial profiling to reveal interactions within the tumor microenvironment of left- and right-sided disease [58].

Among the proteins identified as differentially expressed between RCRC and LCRC (Table 1), PRAC1, a small nuclear protein, has been frequently referenced to be of higher expression in LCRC [49, 51–53, 59]. However, given that PRAC1 has been reported to not be correlated with patient outcome in either side [60], and its repression in the right side is expected to be the result of hypermethylation [49], further work is required to determine whether PRAC1 is truly oncogenic in the left side of the colon, or just exclusively expressed in the region. *PRAC1* has also been speculated to be co-transcribed with the *HOXB13* gene, which itself has been reported as being of higher expression in LCRC [54, 60, 61]. Interestingly, although of higher expression in LCRC, *HOXB13* expression was observed to be associated with improved patient outcomes in RCRC only, where it was distinct from LCRC in exhibiting differential expression from normal tissue specimens [60]. *HOXB13* was also found to be tumor suppressive in vitro [60], further highlighting its potential as a candidate gene for targeted therapeutic intervention.

Additional members of the HOX family of transcription factors, *HOXC4*, *HOXC6* and *HOXC8*, have also been found to be differentially expressed between RCRC and LCRC, although unlike *HOXB13*, they demonstrate greater expression in disease affecting the right side of the colon [51–54, 59]. Like *PRAC1*, *HOXC6* specifically is frequently reported as one of the most differentially expressed genes [51–54, 59] (Table 1), and, notably, has been linked to epithelial-to-mesenchymal transition (EMT) and tumor proliferation in various malignancies, including CRC [62–66]. In cervical cancer, experimental silencing of *HOXC6* with small interfering RNA (siRNA) was seen to inhibit EMT through the TGF-b signaling pathway [62], however in CRC, *HOXC6*-related proliferation [63] and EMT [64] were found to require mTOR and Wnt signaling, respectively, suggesting a multifaceted, and potentially plastic, role of *HOXC6* in disease progression. Furthermore, *HOXC6* expression has also been related to the cellular composition of the tumor microenvironment (TME), with immune infiltration higher in patients with increased *HOXC6* expression [66]. Although such infiltration is characteristic of RCRC generally [67], other RCRC-associated traits, such as high expression of immune checkpoint markers [66], were similarly observed in high *HOXC6*-expressing glioma cells, which, strikingly, suggests that *HOXC6* may be supporting the aggressive pathogenesis of RCRC through pan-cancer mechanisms [63].

In further relation to the TME, cancer-associated fibroblasts (CAFs) have also been implicated in the expression of *HOXC6*. Specifically, CAF-derived extracellular vesicles containing the lncRNA SNHG3 have been found to increase the expression of *HOXC6*, inciting greater tumor proliferation [68]. Alongside this particular study, which emphasized the role of the mesenchymal niche in supporting CRC growth, other work has also reported the differential expression of various lncRNAs between RCRC and LCRC [50, 51, 55, 59]. However, the function of many has yet to be determined, thus representing the need for further investigation. Similarly, miRNAs are also commonly reported as differentially expressed, including the findings described by Eneh and colleagues [69], but again require more detailed characterization and experimental validation [70].

Alongside *HOXC6*, whereby gene silencing was seen to increase CRC cell sensitivity to irinotecan [66], a recent study has shown that knockdown of *FOXD1*, another transcription factor also of higher expression in RCRC [53, 54], increases sensitivity to oxaliplatin both in vitro and in vivo [71]. Although this was cited as relative to increased cell stemness and the exact mechanism not determined, *FOXD1* has otherwise been described as a promoter of CRC cell proliferation through regulation of the polo-like kinase 2 protein [72], which itself has been associated with chemoresistance in CRC [73]. While transcription factors have historically been difficult targets for novel cancer therapies [74], they nevertheless provide the opportunity for more personalized patient prognostication and prediction of therapy response.

Such is also the case for other nucleic acid binding proteins, including SATB2 [50, 75, 76], which facilitates transcription through the induction of chromatin loops, and whose loss is associated with poor prognosis and right sidedness. Similarly, the expression of R-loop binding proteins, which help to maintain genomic stability, has been observed to cluster into categories of low and high expression, with low expression again associated with poor prognosis and right sidedness [77]. Interestingly, the loss of *ELAVL2*, whose protein binds and stabilizes mRNA, is also associated with right sidedness [50, 53], as well as aggressive tumor behavior in other cancer types [78, 79]. Whilst dysregulation of nucleic acid binding proteins and other transcriptional regulators is not a feature of RCRC, genes associated with aggressive disease are frequently reported to be of significant difference between right and left tumor sites, and as demonstrated, are more commonly associated with right-sided primary tumor location.

**Table 1:** Differentially expressed genes related to transcriptional regulation

	Gene	Side^a^	Function	Reference
Epigenetic modifiers	*FIP1L1*	RCRC	mRNA polyadenylation	[50]
	*KMT5C*	LCRC	Histone methyltransferase	[80]
	*PRAC1*	LCRC	Unknown, associated with hypermethylation	[49, 51–53, 59]
Nucleic acid binding proteins	*HNRNPA1*	RCRC	Splicing factor/RNA-binding protein	[50]
	*HNRNPC*	RCRC	Splicing factor/RNA-binding protein	[50]
	*KHDRBS1*	RCRC	Splicing factor/RNA-binding protein	[50]
	*RBM25*	RCRC	Splicing factor/RNA-binding protein	[50]
	*SRRM1*	RCRC	Splicing factor/RNA-binding protein	[50]
	*ZC3H12C*	RCRC	Ribonuclease	[55]
	*ESRP1*	LCRC	Splicing factor/RNA-binding protein	[50]
	*ELAVL2*	LCRC	Splicing factor/RNA-binding protein	[50, 53]
	*FMR1*	LCRC	Splicing factor/RNA-binding protein	[50]
	*KHDRBS3*	LCRC	Splicing factor/RNA-binding protein	[50]
	*SATB2*	LCRC	DNA-binding protein	[50, 75, 81]
	*SRSF6*	LCRC	Splicing factor/RNA-binding protein	[50]
Zinc finger proteins	*CIZ1*	RCRC	Zinc finger	[50]
	*FEZF1*	RCRC	Zinc finger	[53]
	*PLAGL2*	RCRC	Zinc finger	[54]
	*ZNF83*	RCRC	Zinc finger	[50]
Long non-coding RNAs	*AC005186.1*	RCRC	Long non-coding RNA	[51]
	*AC012531.25*	RCRC	Long non-coding RNA	[59]
	*AFAP1-AS1*	RCRC	Long non-coding RNA	[51]
	*LINC00908*	RCRC	Long non-coding RNA	[50]
	*PLEKHA8P1*	RCRC	Long non-coding RNA	[55]
	*RP11-278A23.1*	RCRC	Long non-coding RNA	[55]
	*RP11-45K12.7*	RCRC	Long non-coding RNA	[55]
	*RP11-626H12.2*	RCRC	Long non-coding RNA	[55]
	*RP11-742B18.1*	RCRC	Long non-coding RNA	[55]
	*AC108134.3*	LCRC	Long non-coding RNA	[51]
	*AC108865.1*	LCRC	Long non-coding RNA	[51]
	*CTD-2012K14.8*	LCRC	Long non-coding RNA	[55]
	*CTD-2184C24.2*	LCRC	Long non-coding RNA	[55]
	*RP11-383I23.2*	LCRC	Long non-coding RNA	[59]
	*RP11-51F16.9*	LCRC	Long non-coding RNA	[55]
	*RP11-59D5_B.2*	LCRC	Long non-coding RNA	[55]
	*RP11-680F8.3*	LCRC	Long non-coding RNA	[55]
Transcription factors	*EMX1*	RCRC	Transcription factor	[53]
	*FOXD1*	RCRC	Transcription factor	[53, 54]
	*HOXC4*	RCRC	Transcription factor	[59]
	*HOXC6*	RCRC	Transcription factor	[51–54, 59]
	*HOXC8*	RCRC	Transcription factor	[53, 59]
	*NR1H4*	RCRC	Transcription factor	[54]
	*HOXB13*	LCRC	Transcription factor	[54, 60]
	*MAGEB17*	LCRC	Transcription factor	[51]
	*PTF1A*	LCRC	Transcription factor	[53]

^a^ Side listed is that of higher gene expression

### Tumor metabolism and cell signaling is likely confounded by the side-dependent genomic landscape of CRC

Metabolic reprogramming is associated with CRC growth, progression and chemoresistance, and is dependent on the specific mutational profile of a patient’s tumor. Namely, Wnt, MAPK, PI3K and p53 signaling have all been individually implicated in aberrant metabolic processes [82, 83], with further changes observed in response to chemotherapeutic treatment [84, 85]. While side-associated disease characteristics, such as RCRC-linked *BRAF* mutation, are therefore likely to contribute to observed differences in metabolic gene expression (Table 2), these markers provide a foundation for future investigative and mechanistic research nonetheless. Interestingly, ligands of commonly mutated signaling pathways have been reported to be differentially expressed, including activin A in RCRC [86] and *LEFTY1* in LCRC [54], while signaling antagonists, such as *DKK4* in RCRC [54] and *WIF1* in LCRC [51], were also noted, indicating possibly side-exclusive mechanisms through which these pathways contribute to disease pathogenesis and progression. The genes referenced in these studies also corroborate the diversity of signaling pathways altered in RCRC and LCRC, as mentioned in relation to transcriptional regulators, with Wnt, BMP and TGF-b interactors all highlighted between disease sites [51, 54, 55, 86]. Otherwise, genes such as *FABP1* reinforce the clinicopathological differences existing between RCRC and LCRC, such that *FABP1* is commonly used as a marker of differentiated enterocytes [87, 88] and aligns with the greater differentiation of left-sided tumors [30, 31]. Meanwhile, the upregulation of *TRIM29* in RCRC again aligns with the aggressive nature of right-sided disease, given *TRIM29* has been linked to an increased risk of recurrence and death, exclusively in RCRC [89]. Other studies have also demonstrated the carcinogenic role of *TRIM29* in CRC, with overexpression resulting in increased cell invasion and migration both in vitro and in mouse models [90], in addition to knockdown preventing disease progression [90, 91]. Such was similarly observed in pancreatic ductal adenocarcinoma cells lines, whereby overexpression was also found to promote resistance to the chemotherapeutic agent gemcitabine by increasing DNA synthesis [92], with an additional study also relating *TRIM29* to DNA damage repair [93]. Interestingly, *TRIM29* overexpression has also been demonstrated to resensitize an oxaliplatin-resistant CRC cell line [94], although, given this was related to *TP53* mutant status, of which the TRIM29 protein is not dependent [95] and which is less commonly observed in RCRC [34, 38], further research is necessary to confirm this phenomenon in *TP53* wildtype tumors.

**Table 2:** Differentially expressed genes related to cellular metabolism and signaling

	Gene/protein	Side^a^	Function	Reference
Cellular metabolism	*CA8*	RCRC	Acatalytic carbonic anhydrase	[51]
	*CASP3*	RCRC	Apoptotic protease	[96]
	*CPS1*	RCRC	Carbamoyl phosphate synthetase	[54]
	*LPO*	RCRC	Peroxidase	[55]
	*PLA2G4A*	RCRC	Phospholipase	[97]
	*SPATA18*	LCRC	Mediator of mitochondrial quality	[54]
	*MCT4*	LCRC	Proton pump	[98]
	*FABP1*	LCRC	Fatty acid binding protein	[76]
	*PRKAA2*	LCRC	Protein kinase catalytic subunit	[59]
	*QPRT*	LCRC	Quinolinate catabolism enzyme	[59]
	*RNLS*	LCRC	Amine oxidase	[59]
Drug metabolism	*p130Cas*	RCRC	Substrate of integrin signaling	[99]
	*CYP11A1*	LCRC	Cytochrome P450 monooxygenase	[55]
Protein trafficking	*CHMP7*	RCRC	Multivesicular body protein	[59]
	*METTL11B*	RCRC	Protein methyltransferase catalyst	[55]
	*MOCS1*	RCRC	Activator of molybdenum	[55]
	*PTGS2*	RCRC	Dioxygenase and peroxidase in hormone synthesis	[55]
	*TRIM29*	RCRC	Histone-binding protein involved in DNA repair	[89, 90]
	*PCMTD2*	LCRC	Protein methylation	[59]
	*PCSK1N*	LCRC	Inhibitor of prohormone convertase 1	[51]
	*SMPD1*	LCRC	Sphingomyelin phosphodiesterase	[55]
Cell signaling	Activin A	RCRC	TGF-b ligand	[86]
	*CTSE*	RCRC	Peptidase	[54]
	*DKK4*	RCRC	Wnt inhibitor	[54]
	*DUSP4*	RCRC	Phosphatase	[54]
	Follistatin	RCRC	Activin antagonist	[86]
	*GABRP*	RCRC	GABA receptor subunit	[51]
	*TCN1*	RCRC	Vitamin B12 binding protein	[54]
	*DAND5*	LCRC	BMP agonist	[55]
	*DUSP2*	LCRC	Phosphatase	[100]
	*GNG4*	LCRC	G-protein coupled receptor subunit	[101]
	*LEFTY1*	LCRC	TGF-b ligand	[54]
	*REG1A*	LCRC	Secreted regenerating protein	[54]
	*WIF1*	LCRC	Wnt inhibitor	[51]

^a^ Side listed is that of higher gene expression

### Side-related genes involved in tumor cell architecture are linked to disease pathogenesis and therapy resistance

The intricate interplay of cell adhesion and structural proteins within a tumor determines its ability to proliferate and progress within the greater microenvironmental context. Genes encoding structural proteins, such as *CNTRL* and *MAST1*, are among those reported as differentially expressed between RCRC and LCRC (Table 3), however most have yet to be experimentally validated beyond the context of RNA sequencing [50, 59]. Nevertheless, several have been linked to a cancer setting in other studies, including *NRP1* [102], *KRT23* [103], *SPRR1A* [104] and *MAST1* [105]. Notably, elevated *MAST1* expression has been implicated in platinum-based chemotherapy resistance, with *MAST1* inhibition shown to restore cisplatin sensitivity in vitro [105].

Additionally, a cohort of cell membrane proteins that contribute to tumor structure, as well as to intracellular and intercellular signaling, have also been reported to exhibit differential expression between RCRC and LCRC. Of note, *LY6G6D*, which encodes a membrane protein of the major histocompatibility complex (MHC) family, has been reported to be of higher expression in LCRC [53, 54], as has related gene *XXbac-BPG32J3.19* [53]. *LY6G6D*, however, has been demonstrated to be selectively expressed in MSS CRC cells [106], meaning that the increased expression observed in LCRC is likely attributed to the predominance of MSS disease within the left side. Given *LY6G6D* has also been implicated in the immune resistance that is characteristic of MSS disease [107], targeting *LY6G6D* may provide a novel opportunity for the treatment of MSS CRC patients.

The expression of certain membrane markers, when linked to poor prognosis, are also often associated with right-sided tumor location. Such is the case with *MUC12*, a membrane glycoprotein gene related to barrier function, whereby low expression has been related to poor disease-free survival outcomes [108], and which has been reported as of lower expression in RCRC [52, 54]. Similarly, a reduction in *DAB2* expression, which encodes an endocytosis-regulating transmembrane protein, has been related with disease progression [109] and has recently been shown to be lowly expressed in high-grade and right-sided tumors [110]. Interestingly, *DAB2* has also emerged as a potential contributor to immune suppression. Like *LY6G6D*, *DAB2* is associated with the low neoantigen load characteristic of LCRC and also interferes with tissue-resident immune cell signaling [111]. This suggests that varying levels of *DAB2* expression, both high and low, may play a role in distinguishing between right-sided and left-sided disease.

**Table 3:** Differentially expressed genes related to cell membrane and structure

	Gene	Side^a^	Function	Reference
Cell membrane	*C17orf78*	RCRC	Cell membrane protein	[51]
	*HM13*	RCRC	Endoplasmic reticulum membrane protein	[50]
	*SLITRK6*	RCRC	Cell membrane protein	[51]
	*CLDN8*	LCRC	Tight junction protein	[54]
	*DAB2*	LCRC	Cell membrane protein	[110]
	*GBAS*	LCRC	Cell membrane protein	[50]
	*LY6G6D*	LCRC	Cell membrane protein	[53, 54]
	*MUC12*	LCRC	Membrane glycoprotein	[52, 54]
	*NKPD1*	LCRC	Cell membrane protein	[55]
	*TMEM63C*	LCRC	Cation membrane transporter	[59]
	*TMUB2*	LCRC	Cell membrane protein	[50]
Cell structure	*CNTRL*	RCRC	Centriole structural protein	[59]
	*MAST1*	RCRC	Microtubule associated protein	[50]
	*NRP1*	RCRC	Cell migratory protein	[50]
	*SPRR1A*	RCRC	Peptide cross-linking protein	[51]
	*ABI1-TSV-11*	LCRC	Adaptor protein	[112]
	*KRT23*	LCRC	Keratin protein	[54]
	*MYOM3*	LCRC	Structural protein	[59]
	*SPAG16*	LCRC	Structural protein	[59]
Extracellular matrix	*TIMP1*	RCRC	Metallopeptidase inhibitor	[113]
	*SPARCL1*	LCRC	Secreted matricellular glycoprotein	[114]

^a^ Side listed is that of higher gene expression

### Differing immune and microbial profiles define RCRC and LCRC, even when accounting for microsatellite instability

**Fig. 2:**
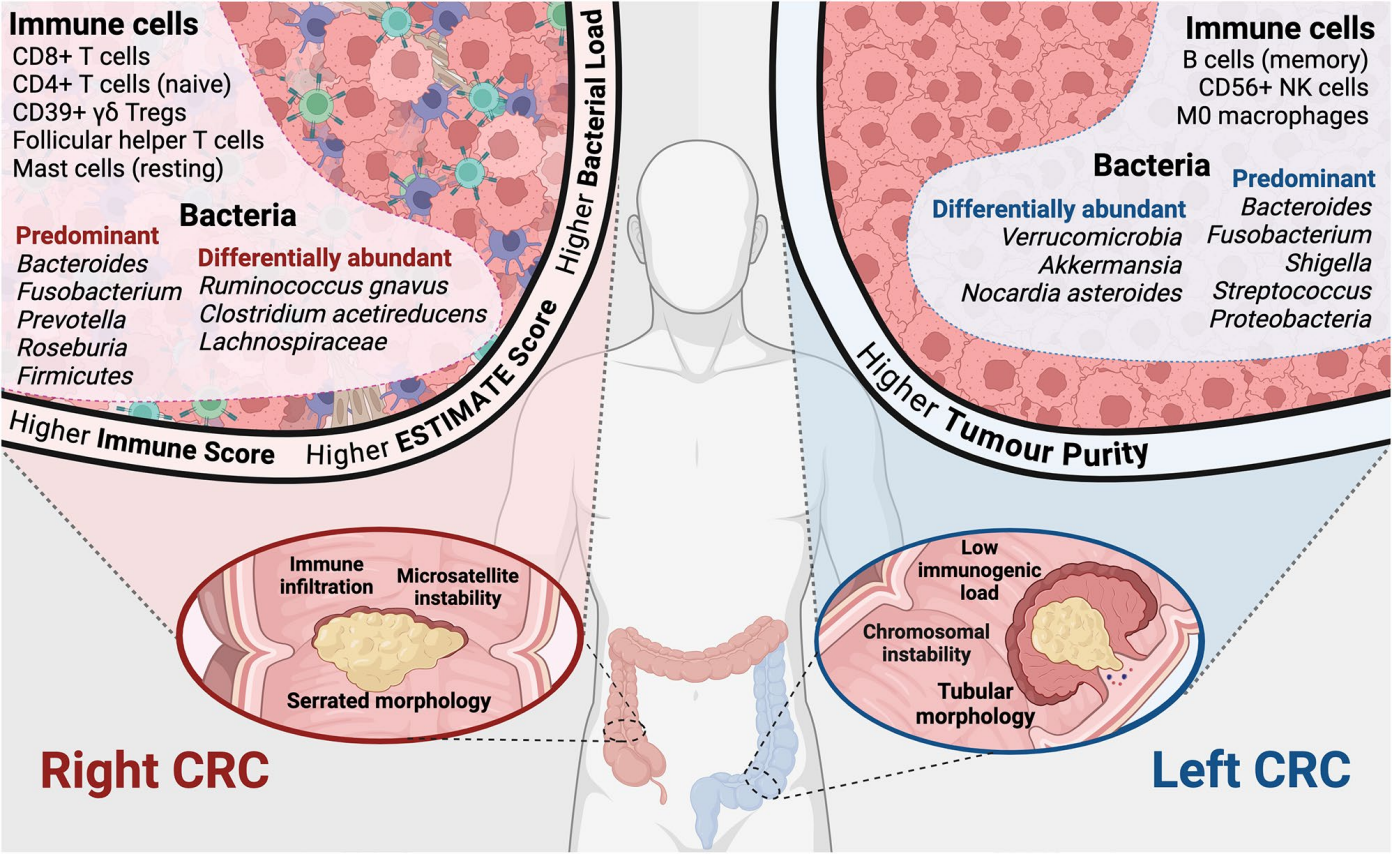
Sidedness-associated differences in immune and bacterial abundance within the tumor microenvironment. While LCRC is associated with disease characterized by polypoid adenocarcinomas and chromosomal instability, RCRC is conversely associated with serrated tumor morphology and MSI. On account of the MSI phenotype, immune infiltration is often reported as higher in RCRC, as is the immune and stromal conjugate ESTIMATE score, while tumor purity is accordingly greater in left-sided disease. Even so, certain subsets of immune cells, including natural killer (NK) cells and memory B cells, have been reported as more abundant in LCRC. Meanwhile, the predominant microbiota of RCRC and LCRC tumors have been presented as distinct, with the often RCRC-associated *Fusobacterium* reported to also dominate the LCRC flora, in addition to the description of various other bacteria as differentially abundant between sides. Data were retrieved from and based on references [34, 55, 59, 67, 97, 117, 124, 127, 129, 130, 137]

As a result of the higher mutational burden of RCRC, usually incited by MSI and deficient mismatch repair (MMR) proteins, RCRC is often associated with greater immune infiltration and improved response to immunotherapies, such as anti-PD-1 [65, 115]. So, while left-sided disease has been found to be of greater tumor purity, RCRC has instead been correlated with a greater immune score, including higher counts of a variety of lymphoid cells [55, 116, 117] (Fig. 2). Similarly, the expression of immune-checkpoint and human leukocytic antigen (HLA) related genes has also been found to be increased in right-sided disease [55], alongside various other immune-related markers (Table 4). In explication of these associations, a recent study utilizing an existing scRNAseq dataset revealed two side-dependent meta-programs which influence the immune landscape of RCRC and LCRC [118]. Specifically, they define a LCRC-associated proliferation stemness (PS) meta-program linked to cell cycle progression genes and stem cell identity, and an RCRC-associated immune secretory (IS) meta-program linked to the expression of MHC class II molecules, including HLA genes. While the PS meta-program was associated with activated regulatory T cells (Tregs) in LCRC and the IS meta-program associated with attenuated CD161-positive CD8-positive T cells in RCRC, for example, further analysis is required to conclude that these programs are indeed side-dependent after accounting for MSI-related differences known to exist between sides.

Clinically, the prognostic value of specific immune cells and their relative proportions also differ between RCRC and LCRC. For instance, while activated tissue resident memory T cells have been shown to be predictive of good prognosis in LCRC patients, the same cells indicate poor prognosis in RCRC [119]. Similarly, the ratio of lymphocytes to monocytes has been found to be higher in LCRC, although this is only predictive of prolonged survival in RCRC patients [120]. Interestingly, Foxp3, a marker of Tregs [121], has been reported to correlate with poor prognosis in LCRC patients only [122], while at the same time, CD39-positive γδ T cells, a subset of Tregs [123], have been shown to be increased in right-sided disease [97], indicating differing mechanisms of immune suppression. Specifically, these CD39-positive γδ Tregs have been associated with the activation of the phospholipase A2-IVA/arachidonic acid (PLA2G4A/AA) metabolic pathway exclusively in RCRC [97]. Meanwhile, CD4-positive T cells, including populations of Tregs, have been shown to be more functional in left-sided disease, also in part due to metabolic factors [124]. This further suggests a difference in immune activity between sides that extends beyond microsatellite stability and mismatch repair status.

Also active within the TME, mesenchymal niche cells, such as CAFs, have been determined to contribute to CRC carcinogenesis and progression [125], but the difference between RCRC and LCRC populations remains to be elucidated [126]. Differences in the microbiota of those with RCRC and LCRC, however, has been studied more thoroughly [127, 128], although contention still remains when defining side-dominant genera. For instance, where *Fusobacterium* has been linked to poor prognosis and RCRC mucosa by Jin et al. [129], it has also been shown to predominate the fecal microbiota in LCRC by Miyake et al. [130]. Meanwhile, studies by Du et al. have described an association between the presence of *Fusobacterium* and a favorable prognosis [53]. In their recent large-scale analysis of colorectal tumors, Cornish et al. clarify side-dominant genera, particularly *Fusobacterium*, to not only differ between sides, but also between MSI and MSS tumors [34]. While their cohort of left-sided MSI tumors was of insufficient size for comparison, the proportion of *Fusobacterium* in right-sided MSI tumors was significantly higher than in right-sided MSS tumors, with both of significantly greater proportion than MSS left-sided tumors, although *Fusobacterium* remained among the most predominant genera in all tumors. Meanwhile, the proportion of *Akkermansia* bacteria was found to be greater in left-sided tumors, as previously reported by Kolisnik et al. [59].

To relate this to prognosis, various side-related microbial genera have been associated with chemotherapy response in pre-clinical studies. Specifically, RCRC-associated *Fusobacterium nucleatum* has been linked to 5-fluorouracil (5-FU) and oxaliplatin chemoresistance through toll-like receptor 4 (TLR4)-mediated autophagy [131], with *BIRC3* later implicated in the same mechanism of 5-FU resistance [132]. Meanwhile, *Fusobacterium nucleatum* has also been observed to promote anti-PD-1 immunotherapy resistance through the release of succinic acid, a molecule which was shown to reduce CD8-positive T cell count in a murine model [133]. Otherwise, *Prevotella*, which has also been associated with RCRC, has similarly been observed to contribute to 5-FU-based chemotherapy resistance and disease progression [134, 135], while, on the other hand, LCRC-linked *Akkermansia muciniphila* has been evidenced to potentiate the efficacy of 5-FU and oxaliplatin [136] and RCRC-linked *Firmicutes* [137] has been associated with improved patient survival [138].

In translation to the clinic, various studies have assessed the effect of probiotics and fecal microbiota transplants (FMT) on the efficacy of standard adjuvant therapies [139], however these have attempted to restore microbial homeostasis within the gut, rather than target specific bacterial populations. The vastly reduced microbial load of metastatic disease [34], in addition to difficulty in targeting microbiota at sites of metastasis [140], presents the ongoing challenge of how best to modulate bacteria beyond the colon. The higher bacterial load of RCRC [34], which mirrors the increased homeostatic abundance of bacteria in the right colon [141], presents the attractive prospect of antimicrobial agents being used to bolster anti-tumor efficacy [142], but such treatment poses the risk of off-target disruption of an already diminished colonic flora [139, 143]. Nevertheless, the complex interplay between tumor-promoting and tumor-protective microbiota, and their joint influence on chemosensitivity, suggests microbial modulation as a potential therapeutic opportunity for both RCRC and LCRC [144], with further research to determine the feasibility of clinical application.

**Table 4:** Differentially expressed genes related to tumor microenvironment

	Marker	Side^a^	Function		Reference
Immune-related genes	*CXCL11*	RCRC	Chemokine	[54]	
	*CXCL17*	RCRC	Chemokine	[51]	
	*GZMA*	RCRC	Granzyme A	[59]	
	*HLA-B*	RCRC	Leukocytic antigen	[55]	
	*HLA-C*	RCRC	Leukocytic antigen	[55]	
	*HLA-D*	RCRC	Leukocytic antigen	[55]	
	*HLA-DMA*	RCRC	Leukocytic antigen	[55]	
	*HLA-DOA*	RCRC	Leukocytic antigen	[55]	
	*HLA-DOB*	RCRC	Leukocytic antigen	[55]	
	*HLA-DPA1*	RCRC	Leukocytic antigen	[55]	
	*HLA-DPA3*	RCRC	Leukocytic antigen	[55]	
	*HLA-DPB1*	RCRC	Leukocytic antigen	[55]	
	*HLA-DQB1*	RCRC	Leukocytic antigen	[55]	
	*HLA-DQB2*	RCRC	Leukocytic antigen	[55]	
	*HLA-DRA*	RCRC	Leukocytic antigen	[55]	
	*HLA-DRB1*	RCRC	Leukocytic antigen	[55]	
	*HLA-DRB5*	RCRC	Leukocytic antigen	[55]	
	*HLA-DRB6*	RCRC	Leukocytic antigen	[55]	
	*HLA-E*	RCRC	Leukocytic antigen	[55]	
	*HLA-F*	RCRC	Leukocytic antigen	[55]	
	*HLA-K*	RCRC	Leukocytic antigen	[55]	
	*HLA-V*	RCRC	Leukocytic antigen	[55]	
	*IGLV3-19*	RCRC	Immunoglobulin lambda variable	[51]	
	*SOCS6*	RCRC	Suppressor of cytokine signaling	[59]	
	*ULBP2*	RCRC	MHC1 related natural killer cell recruiter	[51]	

^a^ Side listed is that of higher expression

## Conclusions

Recent studies evaluating the differential gene expression of RCRC and LCRC tumors have reported a variety of targets encompassing a range of cellular functions. In this review, we summarized genes, proteins and cell types that represent significant variation between sides, and highlighted candidates for further investigation, including those that have been linked to disease progression and therapy resistance. Importantly, it is clear that differentially expressed genes associated with aggressive disease characteristics and poor patient outcomes are more often associated with RCRC. However, given differences in the distribution of key driver mutations between sides, as well as the varied impact of microsatellite instability and DNA mismatch repair protein deficiency, this review also highlights the need for added comparisons which extend beyond the fundamental anatomical differences of the colon. Additionally, experimental validation of gene targets identified through RNA sequencing will assist in confirming both their association and role in mediating differences in patient prognosis, with innovative spatial profiling to enable such investigation in the context of side-dependent microenvironmental niches. These future studies will likely inform the development of novel side-specific therapy options and improved patient outcomes.

## Data Availability

No datasets were generated or analysed during the current study.

## Abbreviations

5-FU: 5-flurouracil
APC: Adenomatous polyposis coli
BMP: Bone morphogenetic protein
BRAF: v-raf murine sarcoma viral oncogene homolog B1
CAF: Cancer-associated fibroblast
CDX: Caudal-related homeobox
CMS: Consensus molecular subtype
CNTRL: Centriolin
CRC: Colorectal cancer
CTLA-4: Cytotoxic T-lymphocyte-associated protein 4
DAB2: Disabled-2
DKK4: Dickkopf-4
EGFR: Epidermal growth factor receptor
ELAVL2: ELAV like RNA binding protein 2
EMT: Epithelial-mesenchymal transition
ESTIMATE: Estimation of stromal and immune cells in malignant tumor tissues using expression data
FABP1: Fatty acid binding protein 1
FMT: Fecal microbiota transplant
FOXD1: Forkhead box D1
HER2: Human epidermal growth factor 2
HLA: Human leukocyte antigen
HOX: Homeobox
ID2: Inhibitor of DNA binding 2
IDI1: Isopentenyl-diphosphate delta isomerase 1
IS: Immune secretory
KRAS: Kristen rat sarcoma viral oncogene homolog
KRT23: Keratin 23
LCRC: Left colorectal cancer
LEFTY1: left-right determination factor 1
lncRNA: Long non-coding RNA
LY6G6D: Lymphocyte antigen 6 complex
MAPK: Mitogen-activated protein kinase
MAST1: Microtubule associated serine/threonine kinase 1
MHC: Major histocompatibility complex
miRNA: Micro RNA
MMR: Mismatch repair
mRNA: Messenger RNA
MSI: Microsatellite instable
MSS: Microsatellite stable
mTOR: Mammalian target of rapamycin
MUC12: Mucin 12
NK: Natural killer
NRP1: Neuropilin 1
PD-1: Programmed cell death protein 1
PI3K: Phosphatidylinositol-3 kinase
PIK3CA: Phosphatidylinositol-4,5-bisphosphate 3-kinase catalytic subunit alpha
PLA3G4A/AA: Phospholipase A2-IVA/arachidonic acid
PRAC1: Prostate cancer susceptibility candidate 1
PS: Proliferation secretory
RCRC: Right colorectal cancer
SATB2: Special AT-rich sequence-binding protein 2
scRNAseq: Single-cell RNA-sequencing
siRNA: Small interfering RNA
SNHG3: Small nucleolar RNA host gene 3
SPRR1A: Small proline rich protein 1 A
TGF: Transforming growth factor
TLR4: Toll-like receptor 4
TME: Tumor microenvironment
TP53: Tumor protein p53
Tregs: Regulatory T cells
TRIM29: Tripartite motif-containing 29 protein
VEGF: Vascular endothelial growth factor
WIF1: Wnt inhibitory factor 1
